# Bis(2,4-dinitro­phen­yl)sulfane

**DOI:** 10.1107/S1600536812041803

**Published:** 2012-10-13

**Authors:** M Buvaneswari, D Kalaivani, M Nethaji

**Affiliations:** a PG and Research Department of Chemistry, Seethalakshmi Ramaswami College, Tiruchirappalli 620 002, Tamil Nadu, India; b Department of Inorganic and Physical Chemistry, Indian Institute of Science, Bangalore 560 012, India

## Abstract

In the title compound, C_12_H_6_N_4_O_8_S, the dinitro­phenyl rings subtend an angle of 78.46 (13) °. In the crystal, mol­ecules are linked by weak C—H⋯O hydrogen bonds leading to the formation of a two-dimensional network lying parallel to the *bc* plane.

## Related literature

For applications of bis­(2,4-dinitro­phen­yl)sulfane, see: Nakadate *et al.* (1964 ▶); Alekhina *et al.* (1978 ▶); Parihar *et al.* (1971 ▶); Evans & Kinnard (1983 ▶); Andricopulo *et al.* (2006 ▶). For related syntheses, see: Pesin *et al.* (1963 ▶); Joshi & Mathur (1963 ▶); Obata *et al.* (1966 ▶); Stepanov *et al.* (1974 ▶); Davydov & Beletskaya (2003 ▶). For our previous work to synthesize new substituted barbiturates, see: Manickkam & Kalaivani (2011 ▶); Rajamani & Kalaivani (2012 ▶).
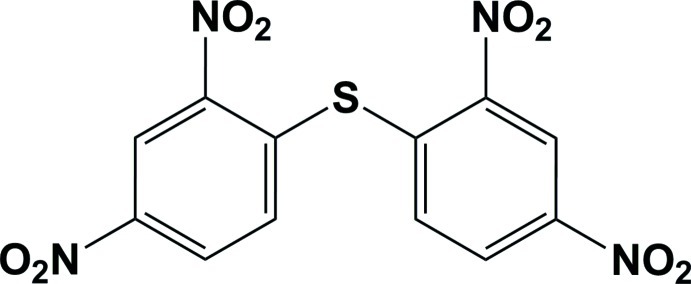


## Experimental

### 

#### Crystal data

C_12_H_6_N_4_O_8_S
*M* *_r_* = 366.27
Monoclinic, 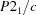

*a* = 9.9428 (12) Å
*b* = 7.3693 (9) Å
*c* = 19.743 (2) Å
β = 95.525 (8)°
*V* = 1439.9 (3) Å^3^
*Z* = 4
Mo *K*α radiation
μ = 0.28 mm^−1^
*T* = 296 K
0.15 × 0.06 × 0.03 mm

#### Data collection

Bruker SMART APEX CCD diffractometer
Absorption correction: multi-scan (*SADABS*; Bruker, 2004 ▶) *T*_min_ = 0.958, *T*_max_ = 0.991
15342 measured reflections
2949 independent reflections
1651 reflections with *I* > 2σ(*I*)
*R*_int_ = 0.070

#### Refinement

*R*[*F* ^2^ > 2σ(*F* ^2^)] = 0.046
*wR*(*F* ^2^) = 0.110
*S* = 0.99
2949 reflections
226 parameters
H-atom parameters constrained
Δρ_max_ = 0.21 e Å^−3^
Δρ_min_ = −0.19 e Å^−3^

### 

Data collection: *APEX2* (Bruker, 2004 ▶); cell refinement: *SAINT-Plus* (Bruker, 2004 ▶); data reduction: *SAINT-Plus*; program(s) used to solve structure: *SIR92* (Altomare *et al.*, 1993 ▶); program(s) used to refine structure: *SHELXL97* (Sheldrick, 2008 ▶); molecular graphics: *ORTEP-3* (Farrugia, 2012 ▶) and *Mercury* (Macrae *et al.*, 2008 ▶); software used to prepare material for publication: *WinGX* (Farrugia, 2012 ▶).

## Supplementary Material

Crystal structure: contains datablock(s) global, I. DOI: 10.1107/S1600536812041803/su2502sup1.cif

Structure factors: contains datablock(s) I. DOI: 10.1107/S1600536812041803/su2502Isup2.hkl

Supplementary material file. DOI: 10.1107/S1600536812041803/su2502Isup3.cml

Additional supplementary materials:  crystallographic information; 3D view; checkCIF report

## Figures and Tables

**Table 1:** Hydrogen-bond geometry (Å, °)

*D*—H⋯*A*	*D*—H	H⋯*A*	*D*⋯*A*	*D*—H⋯*A*
C3—H3⋯O8^i^	0.93	2.54	3.080 (3)	117
C6—H6⋯O7^ii^	0.93	2.49	3.364 (3)	156
C11—H11⋯O2^iii^	0.93	2.57	3.489 (3)	168

Symmetry codes: (i) 
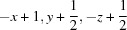
; (ii) 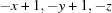; (iii) 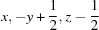.

**Fig. 1.**
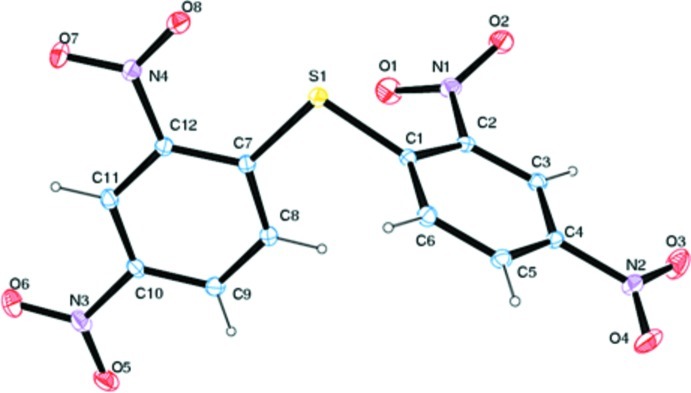
A view of the molecular structure of the title molecule, with the atom numbering. Displacement ellipsoids are drawn at the 30% probability level.

**Fig. 2.**
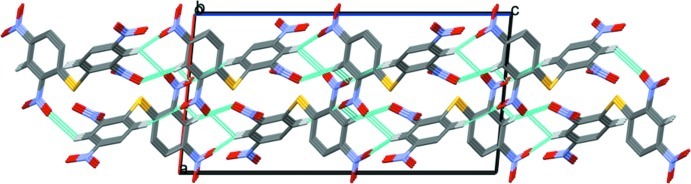
A view along the b axis of the crystal packing of the title compound. The C-H···O hydrogen bonds are shown as dashed cyan lines (H atoms not involved in these interactions have been omitted for clarity; see Table 1 for details).

## supplementary crystallographic information

### Comment

The title sulfur containing organic compound is a biologically active molecule (Andricopulo *et al.*, 2006). It has been utilized in many clinical trials (Nakadate *et al.*, 1964; Alekhina *et al.*, 1978) and also employed in the synthesis of a number of other important organic molecules (Parihar *et al.*, 1971; Evans & Kinnard, 1983). Several methods have been reported for the synthesis of the title molecule (Pesin *et al.*, 1963; Joshi & Mathur, 1963; Obata *et al.*, 1966; Stepanov *et al.*, 1974; Davydov & Beletskaya, 2003). In continuation of our previous work to synthesize new substituted barbiturates when 2-thiobarbituric acid is used (Manickkam & Kalaivani, 2011; Rajamani & Kalaivani, 2012) , the title molecule was obtained crystallizing out as the product through a new synthetic route.

In the title molecule (Fig. 1) the two phenyl rings are inclined to one another by 78.46 (13) °.

In the crystal, there are a number of weak C—H···O hydrogen bonds linking the molecules to form a two-dimensional network lying parallel to the bc plane (Table 1 and Fig. 2).

### Experimental

Analytical grade 1-chloro-2,4-dinitrobenzene (2.02 g, 0.01 mol) dissolved in ethanol (20 ml) and 2-thiobarbituricacid (1.44 g, 0.01 mol) dissolved in ethanol (20 ml) were mixed. Triethylamine (5 g,0.05 mol) was then added and the mixture was shaken well for 5–6 hrs. On standing yellow crystals came out from the solution after 5 days. The crystals were filtered and washed well with ether to remove the unreacted reactants and then with a small amount of absolute alcohol. The crystals were recrystallized from ethanol (M.pt: 467 K; yield: 70%). Good quality single crystals for X-ray diffraction studies were obtained by slow evaporation of a solution in ethanol at room temperature.

### Refinement

All the H atoms were included in calculated positions and treated as riding: C—H = 0.93 Å with U_iso_(H) = 1.2U_eq_(C).

### Crystal data

**Table:**

C_12_H_6_N_4_O_8_S	*F*(000) = 744
*M**_r_* = 366.27	*D*_x_ = 1.690 Mg m^−^^3^
Monoclinic, *P*2_1_/*c*	Mo *K*α radiation, λ = 0.71073 Å
Hall symbol: -P 2ybc	Cell parameters from 687 reflections
*a* = 9.9428 (12) Å	θ = 2.5–26.0°
*b* = 7.3693 (9) Å	µ = 0.28 mm^−^^1^
*c* = 19.743 (2) Å	*T* = 296 K
β = 95.525 (8)°	Needle, yellow
*V* = 1439.9 (3) Å^3^	0.15 × 0.06 × 0.03 mm
*Z* = 4	

### Data collection

**Table:**

Bruker SMART APEX CCD diffractometer	2949 independent reflections
Radiation source: fine-focus sealed tube	1651 reflections with *I* > 2σ(*I*)
Graphite monochromator	*R*_int_ = 0.070
ω scans	θ_max_ = 26.4°, θ_min_ = 2.1°
Absorption correction: multi-scan (*SADABS*; Bruker, 2004)	*h* = −10→12
*T*_min_ = 0.958, *T*_max_ = 0.991	*k* = −9→9
15342 measured reflections	*l* = −24→24

### Refinement

**Table:**

Refinement on *F*^2^	Primary atom site location: structure-invariant direct methods
Least-squares matrix: full	Secondary atom site location: difference Fourier map
*R*[*F*^2^ > 2σ(*F*^2^)] = 0.046	Hydrogen site location: inferred from neighbouring sites
*wR*(*F*^2^) = 0.110	H-atom parameters constrained
*S* = 0.99	*w* = 1/[σ^2^(*F*_o_^2^) + (0.0462*P*)^2^] where *P* = (*F*_o_^2^ + 2*F*_c_^2^)/3
2949 reflections	(Δ/σ)_max_ < 0.001
226 parameters	Δρ_max_ = 0.21 e Å^−^^3^
0 restraints	Δρ_min_ = −0.19 e Å^−^^3^

### Special details

**Table:**

Geometry. Bond distances, angles etc. have been calculated using the rounded fractional coordinates. All su's are estimated from the variances of the (full) variance-covariance matrix. The cell esds are taken into account in the estimation of distances, angles and torsion angles
Refinement. Refinement of *F*^2^ against ALL reflections. The weighted *R*-factor *wR* and goodness of fit *S* are based on *F*^2^, conventional *R*-factors *R* are based on *F*, with *F* set to zero for negative *F*^2^. The threshold expression of *F*^2^ > 2σ(*F*^2^) is used only for calculating *R*-factors(gt) *etc*. and is not relevant to the choice of reflections for refinement. *R*-factors based on *F*^2^ are statistically about twice as large as those based on *F*, and *R*- factors based on ALL data will be even larger.

### Fractional atomic coordinates and isotropic or equivalent isotropic displacement parameters (Å^2^)

**Table:**

	*x*	*y*	*z*	*U*_iso_*/*U*_eq_	
S1	0.45894 (8)	0.54204 (10)	0.14730 (4)	0.0467 (3)	
O1	0.3487 (3)	0.3356 (3)	0.26264 (12)	0.0808 (10)	
O2	0.4165 (2)	0.4985 (3)	0.35003 (11)	0.0765 (10)	
O3	0.0912 (3)	1.0117 (4)	0.35223 (14)	0.0914 (11)	
O4	0.0607 (3)	1.1747 (4)	0.26196 (14)	0.0947 (11)	
O5	−0.0437 (3)	0.1541 (3)	−0.05028 (11)	0.0740 (10)	
O6	0.1255 (3)	0.0405 (4)	−0.09656 (12)	0.0925 (11)	
O7	0.5752 (2)	0.1785 (3)	−0.00808 (11)	0.0684 (9)	
O8	0.6174 (2)	0.3770 (3)	0.07036 (11)	0.0624 (8)	
N1	0.3628 (3)	0.4798 (3)	0.29238 (13)	0.0520 (10)	
N2	0.1077 (3)	1.0440 (4)	0.29312 (16)	0.0599 (11)	
N3	0.0775 (3)	0.1350 (4)	−0.05396 (13)	0.0611 (10)	
N4	0.5382 (3)	0.2847 (3)	0.03373 (12)	0.0459 (9)	
C1	0.3436 (3)	0.6771 (3)	0.18964 (13)	0.0367 (9)	
C2	0.3140 (3)	0.6456 (3)	0.25585 (13)	0.0368 (9)	
C3	0.2394 (3)	0.7630 (4)	0.29087 (13)	0.0423 (10)	
C4	0.1913 (3)	0.9165 (4)	0.25738 (14)	0.0401 (10)	
C5	0.2169 (3)	0.9557 (4)	0.19209 (14)	0.0449 (10)	
C6	0.2941 (3)	0.8364 (4)	0.15892 (13)	0.0440 (10)	
C7	0.3467 (3)	0.4152 (3)	0.09096 (12)	0.0371 (9)	
C8	0.2065 (3)	0.4231 (3)	0.09266 (13)	0.0441 (10)	
C9	0.1189 (3)	0.3318 (4)	0.04641 (14)	0.0477 (10)	
C10	0.1710 (3)	0.2295 (4)	−0.00390 (13)	0.0447 (10)	
C11	0.3068 (3)	0.2136 (3)	−0.00716 (13)	0.0465 (10)	
C12	0.3938 (3)	0.3044 (3)	0.04037 (12)	0.0382 (9)	
H3	0.22210	0.73970	0.33550	0.0510*	
H5	0.18280	1.06060	0.17070	0.0540*	
H6	0.31380	0.86290	0.11490	0.0530*	
H8	0.17140	0.49230	0.12620	0.0530*	
H9	0.02610	0.33860	0.04880	0.0570*	
H11	0.34050	0.14300	−0.04070	0.0560*	

### Atomic displacement parameters (Å^2^)

**Table:**

	*U*^11^	*U*^22^	*U*^33^	*U*^12^	*U*^13^	*U*^23^
S1	0.0398 (5)	0.0482 (4)	0.0514 (5)	0.0035 (4)	0.0003 (4)	−0.0120 (3)
O1	0.103 (2)	0.0390 (13)	0.0968 (18)	−0.0003 (13)	−0.0090 (15)	0.0069 (12)
O2	0.0911 (19)	0.0906 (18)	0.0463 (14)	0.0343 (15)	−0.0016 (13)	0.0164 (12)
O3	0.099 (2)	0.103 (2)	0.0792 (18)	0.0258 (16)	0.0441 (16)	−0.0093 (15)
O4	0.088 (2)	0.0925 (19)	0.103 (2)	0.0568 (17)	0.0064 (16)	−0.0047 (16)
O5	0.0647 (18)	0.0820 (17)	0.0701 (16)	−0.0079 (15)	−0.0199 (14)	−0.0082 (12)
O6	0.101 (2)	0.0974 (19)	0.0755 (17)	0.0002 (17)	−0.0092 (15)	−0.0459 (15)
O7	0.0732 (17)	0.0770 (15)	0.0579 (14)	0.0166 (13)	0.0215 (12)	−0.0157 (12)
O8	0.0480 (14)	0.0682 (14)	0.0712 (15)	0.0035 (12)	0.0067 (12)	−0.0111 (12)
N1	0.0514 (17)	0.0513 (17)	0.0532 (17)	0.0087 (13)	0.0042 (14)	0.0107 (13)
N2	0.0426 (17)	0.0652 (18)	0.072 (2)	0.0118 (15)	0.0060 (15)	−0.0144 (16)
N3	0.072 (2)	0.0569 (17)	0.0512 (17)	−0.0049 (17)	−0.0103 (17)	−0.0068 (13)
N4	0.0543 (18)	0.0448 (14)	0.0394 (14)	0.0080 (13)	0.0084 (13)	0.0055 (11)
C1	0.0345 (17)	0.0352 (15)	0.0391 (16)	0.0006 (13)	−0.0030 (13)	−0.0044 (12)
C2	0.0331 (17)	0.0365 (14)	0.0396 (16)	−0.0003 (13)	−0.0033 (13)	0.0030 (12)
C3	0.0343 (17)	0.0539 (17)	0.0385 (16)	−0.0014 (14)	0.0031 (13)	−0.0004 (13)
C4	0.0292 (17)	0.0413 (16)	0.0493 (18)	0.0016 (13)	0.0019 (13)	−0.0092 (13)
C5	0.0441 (18)	0.0392 (15)	0.0500 (18)	0.0066 (15)	−0.0022 (14)	0.0027 (14)
C6	0.0494 (19)	0.0464 (16)	0.0365 (15)	0.0015 (15)	0.0064 (14)	0.0037 (13)
C7	0.0417 (18)	0.0343 (14)	0.0351 (15)	0.0016 (13)	0.0030 (13)	0.0008 (11)
C8	0.046 (2)	0.0450 (17)	0.0411 (16)	0.0004 (14)	0.0040 (14)	−0.0065 (13)
C9	0.0437 (19)	0.0457 (17)	0.0527 (18)	0.0012 (15)	−0.0007 (15)	−0.0013 (14)
C10	0.055 (2)	0.0404 (16)	0.0364 (16)	−0.0002 (15)	−0.0079 (15)	−0.0013 (13)
C11	0.063 (2)	0.0386 (16)	0.0371 (16)	0.0079 (16)	0.0003 (15)	−0.0010 (13)
C12	0.0444 (19)	0.0350 (14)	0.0353 (15)	0.0048 (13)	0.0047 (14)	0.0047 (12)

### Geometric parameters (Å, º)

**Table:**

S1—C1	1.786 (3)	C2—C3	1.370 (4)
S1—C7	1.766 (3)	C3—C4	1.373 (4)
O1—N1	1.216 (3)	C4—C5	1.369 (4)
O2—N1	1.218 (3)	C5—C6	1.374 (4)
O3—N2	1.218 (4)	C7—C8	1.399 (4)
O4—N2	1.212 (4)	C7—C12	1.405 (3)
O5—N3	1.222 (4)	C8—C9	1.376 (4)
O6—N3	1.224 (4)	C9—C10	1.387 (4)
O7—N4	1.220 (3)	C10—C11	1.363 (4)
O8—N4	1.223 (3)	C11—C12	1.386 (4)
N1—C2	1.477 (3)	C3—H3	0.9300
N2—C4	1.478 (4)	C5—H5	0.9300
N3—C10	1.466 (4)	C6—H6	0.9300
N4—C12	1.462 (4)	C8—H8	0.9300
C1—C2	1.387 (4)	C9—H9	0.9300
C1—C6	1.390 (4)	C11—H11	0.9300
			
C1—S1—C7	101.22 (13)	S1—C7—C8	122.26 (19)
O1—N1—O2	124.8 (3)	S1—C7—C12	121.5 (2)
O1—N1—C2	118.0 (2)	C8—C7—C12	116.3 (2)
O2—N1—C2	117.2 (2)	C7—C8—C9	122.2 (2)
O3—N2—O4	124.1 (3)	C8—C9—C10	119.0 (3)
O3—N2—C4	117.9 (3)	N3—C10—C9	119.0 (3)
O4—N2—C4	118.0 (3)	N3—C10—C11	119.6 (2)
O5—N3—O6	123.9 (3)	C9—C10—C11	121.4 (3)
O5—N3—C10	118.0 (3)	C10—C11—C12	118.9 (2)
O6—N3—C10	118.0 (3)	N4—C12—C7	121.3 (2)
O7—N4—O8	122.6 (3)	N4—C12—C11	116.5 (2)
O7—N4—C12	119.2 (2)	C7—C12—C11	122.2 (3)
O8—N4—C12	118.3 (2)	C2—C3—H3	121.00
S1—C1—C2	123.51 (19)	C4—C3—H3	121.00
S1—C1—C6	118.9 (2)	C4—C5—H5	121.00
C2—C1—C6	117.0 (2)	C6—C5—H5	121.00
N1—C2—C1	120.6 (2)	C1—C6—H6	119.00
N1—C2—C3	116.4 (2)	C5—C6—H6	119.00
C1—C2—C3	123.0 (2)	C7—C8—H8	119.00
C2—C3—C4	117.3 (2)	C9—C8—H8	119.00
N2—C4—C3	118.7 (3)	C8—C9—H9	121.00
N2—C4—C5	118.7 (3)	C10—C9—H9	120.00
C3—C4—C5	122.7 (3)	C10—C11—H11	121.00
C4—C5—C6	118.5 (3)	C12—C11—H11	121.00
C1—C6—C5	121.6 (2)		
			
C7—S1—C1—C2	105.6 (2)	C6—C1—C2—C3	0.1 (4)
C7—S1—C1—C6	−83.5 (2)	S1—C1—C6—C5	−172.9 (2)
C1—S1—C7—C8	−6.2 (2)	C2—C1—C6—C5	−1.3 (4)
C1—S1—C7—C12	171.83 (19)	N1—C2—C3—C4	−179.0 (3)
O1—N1—C2—C1	−46.5 (4)	C1—C2—C3—C4	1.0 (4)
O1—N1—C2—C3	133.5 (3)	C2—C3—C4—N2	178.6 (3)
O2—N1—C2—C1	132.4 (3)	C2—C3—C4—C5	−1.0 (4)
O2—N1—C2—C3	−47.7 (4)	N2—C4—C5—C6	−179.7 (3)
O3—N2—C4—C3	3.8 (4)	C3—C4—C5—C6	−0.1 (5)
O3—N2—C4—C5	−176.6 (3)	C4—C5—C6—C1	1.3 (4)
O4—N2—C4—C3	−176.7 (3)	S1—C7—C8—C9	176.4 (2)
O4—N2—C4—C5	2.9 (4)	C12—C7—C8—C9	−1.7 (4)
O5—N3—C10—C9	1.8 (4)	S1—C7—C12—N4	2.1 (3)
O5—N3—C10—C11	−179.0 (3)	S1—C7—C12—C11	−175.55 (18)
O6—N3—C10—C9	−177.6 (3)	C8—C7—C12—N4	−179.7 (2)
O6—N3—C10—C11	1.7 (4)	C8—C7—C12—C11	2.6 (3)
O7—N4—C12—C7	175.5 (2)	C7—C8—C9—C10	−0.5 (4)
O7—N4—C12—C11	−6.8 (3)	C8—C9—C10—N3	−178.9 (3)
O8—N4—C12—C7	−5.0 (3)	C8—C9—C10—C11	1.9 (4)
O8—N4—C12—C11	172.8 (2)	N3—C10—C11—C12	179.7 (2)
S1—C1—C2—N1	−8.8 (4)	C9—C10—C11—C12	−1.0 (4)
S1—C1—C2—C3	171.3 (2)	C10—C11—C12—N4	−179.1 (2)
C6—C1—C2—N1	−179.9 (3)	C10—C11—C12—C7	−1.3 (4)

### Hydrogen-bond geometry (Å, º)

**Table:**

*D*—H···*A*	*D*—H	H···*A*	*D*···*A*	*D*—H···*A*
C3—H3···O8^i^	0.93	2.54	3.080 (3)	117
C6—H6···O7^ii^	0.93	2.49	3.364 (3)	156
C11—H11···O2^iii^	0.93	2.57	3.489 (3)	168

Symmetry codes: (i) −*x*+1, *y*+1/2, −*z*+1/2; (ii) −*x*+1, −*y*+1, −*z*; (iii) *x*, −*y*+1/2, *z*−1/2.
